# Welding Adjacent Layers in Additively Manufactured Polypropylene via Expansion Annealing

**DOI:** 10.1021/acs.macromol.6c00263

**Published:** 2026-04-27

**Authors:** Zoe Gunter, Anthony Griffin, Niyati Tamang, Zhe Qiang

**Affiliations:** School of Polymer Science and Engineering, 5104 University of Southern Mississippi, 118 College Drive, Hattiesburg, Mississippi 39406, United States

## Abstract

Material extrusion additive manufacturing (MEX-AM) provides a promising alternative to traditional manufacturing methods, as it is accessible, cost-effective, and allows for rapid generation of complex structures. However, a critical limitation of MEX-AM is poor interlaminar adhesion between successive layers, which leads to highly anisotropic mechanical properties and weakness in the build (out-of-plane) direction. Particularly for semicrystalline materials, crystallization can occur rapidly upon material deposition, further restricting the successful welding of subsequent filament traces. In this study, we demonstrate a post-print annealing strategy that directly addresses the weak interlayer properties of semicrystalline 3D-printed polymers. Our approach uses a solvent swelling treatment that selectively penetrates and expands the amorphous domains of printed specimens while preserving their semicrystalline structure. This process promotes enhanced chain entanglement, tie-chain formation, and cocrystallization across interlaminar interfaces, leading to significantly improved mechanical performance along the build direction. In a model system of semicrystalline polypropylene (PP), tensile specimens printed in the build direction exhibit an ∼605% increase in elongation at break and an ∼596% increase in toughness upon expansion annealing. This work establishes a promising, generalizable platform for strengthening 3D printed semicrystalline polymers and advancing the performance of MEX-AM parts.

## Graphical abstract

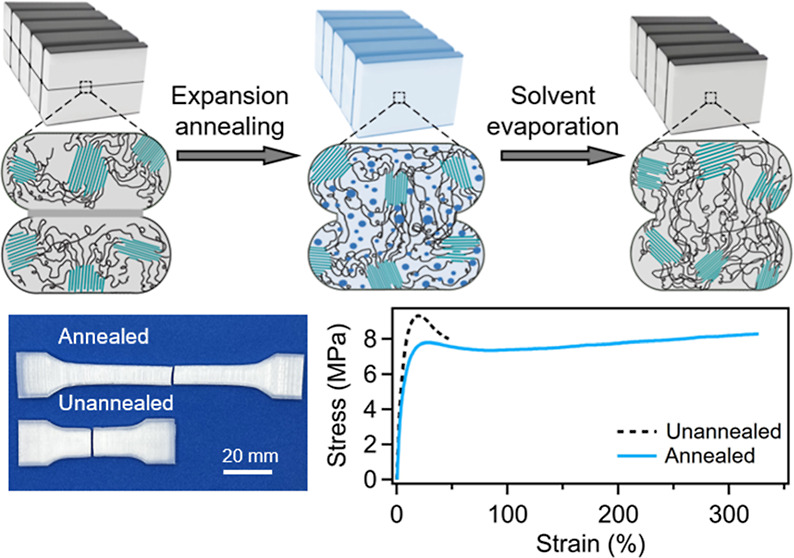

## 1 Introduction

Material extrusion (MEX) is one of the most widely used additive manufacturing (AM) methods owing to its low cost, wide accessibility, and broad materials selection.^1–3^ In this process, molten filament is deposited layer by layer; and for MEX of semicrystalline polymers, the extrudate typically crystallizes quickly upon deposition, thereby fixing the sample shape before the next layer is applied. An intractable challenge of the MEX process is associated with weak interlayer adhesion of printed parts, resulting from restricted chain diffusion across adjacent layers, which yields poor interfacial bonding.^4–11^ Consequently, mechanical properties of MEX-printed parts show a strong dependence on the sample printing orientation. Specifically, tensile specimens printed along the *z*-direction (out-of-plane) are markedly weaker than those printed and examined along the *x* and *y* axes (in-plane).^12,13^ This limitation fundamentally constrains the use of MEX as a drop-in replacement for conventional manufacturing in applications that require isotropic and strong mechanical performance. Thermal annealing methods often fail to improve interlayer adhesion in MEX-printed parts as the required temperatures for accelerating chain diffusion and entanglement formation across adjacent layers are often too high, which can lead to sample destabilization and loss of dimensional accuracy.^14–16^ The trade-off between polymer chain mobility and shape fidelity severely constrains the accessible postprocessing window for materials property improvement.^17^

In recent years, extensive research efforts have been centered on filament design and postprocessing annealing strategies to enhance interlayer bonding and, in turn, improve overall part performance. Example approaches include the addition of reinforcing agents at high loading levels into filaments,^18–24^ incorporation of low molecular weight additives to promote adhesion between layers,^25–28^ introducing dynamic covalent or supramolecular chemistries that enable interlayer cross-linking while preserving sample processability,^29–34^ and engineering core–shell filaments that couple a dimensionally stable core with a diffusible shell.^5,35–41^ Alternatively, advanced annealing methods such as ultrasonic welding and irradiation, or localized microwaving can be employed to improve interlayer adhesion; however, they often require the introduction of susceptors or other additives to enable localized heat.^42–49^ For example, Green et al. coated polylactic acid filament with a carbon nanotube (CNT) layer and, after printing, used microwave irradiation to generate localized heating at layer interfaces, ultimately increasing fracture strength by ∼275%.^49^ While these results are promising, most approaches require specialized equipment or custom filament designs, limiting their broad applicability to commodity filaments produced at scale. Moreover, localized heating enabled by incorporated additives could be sensitive to sample geometry and filler distribution, possibly yielding varied mechanical improvements.

This work introduces a simple, effective approach to interlayer welding of MEX-printed polypropylene (PP) via solvent swelling, referred to as expansion annealing. Our process leverages the semicrystalline nature of PP by immersing printed parts in xylene at elevated temperatures; the solvent selectively swells the amorphous phase, expanding chains and promoting interlayer interactions, while the retention of crystalline domains enables the preservation of part geometry. Upon solvent removal, PP chains recrystallize, strengthening interlayer adhesion and improving out-of-plane mechanical performance without sacrificing dimensional fidelity. As a result, after immersing model tensile specimens in xylene at 50 °C for 150 min, samples exhibited an elongation at a break increase of ∼605% and a toughness increase of ∼596%. These improvements highlight the potential of expansion annealing for serving as a robust, cost-effective method for improving the performance of MEX-printed semicrystalline polymers.

## 2 Experimental Section

### 2.1 Materials Preparation

UltiMaker S series natural polypropylene (PP) filament with a diameter of 2.85 mm was purchased from UltiMaker and used for the material extrusion additive manufacturing (MEX-AM) and compression molding of specimens employed during this study. 3D-printed specimens were created using an UltiMaker S3 printer with an UltiMaker AA 0.4 mm print core. For 3D printing of the PP filament, a nozzle temperature of 215 °C, a bed temperature of 80 °C, and a fan speed of 20% were used. Generally, mechanical specimens were printed with either a 90° or 0° raster angle (relative to the *x*-axis of the printing bed), a line infill geometry, and an infill density of 100% to ensure specimen uniformity and consistency. Additionally, Magigoo adhesive was applied to the bed prior to each print, and a brim was added to each specimen model in the Cura slicing software before printing to allow for sufficient bed adhesion and avoid sample warpage. Generally, a printing speed of 30 mm/s was used for printed specimens; however, printing speeds were modified, and supporting material was utilized for specimens depending on their geometry and print orientation to avoid print failures. All 3D models used for printing throughout this study were either fabricated using Autodesk Fusion or were downloaded directly from htttp://Thigiverse.com. Furthermore, bulk samples of PP were generated via compression molding using a Carver 4386 manual press at a temperature of 190 °C.

### 2.2 Sample Annealing

Expansion annealing of PP specimens was achieved by submerging specimens in a crystallization dish containing xylene (Fisher Scientific, Certified ACS) at varying temperatures (from 25 to 80 °C) with constant stirring and the addition of an external thermocouple to ensure a consistent solvent temperature throughout the immersion vessel. In general, expansion annealing conditions were maintained for all samples, with specimens being immersed for 150 min (well past the point of swelling equilibrium) before being allowed to return to room temperature and removed from the solvent. The samples were then permitted to dry under vacuum for at least 48 h at 40 °C before final measurements were recorded, and subsequent characterization was performed.

### 2.3 General Characterization

To understand the behavior of PP parts upon immersion in xylene at different temperatures, swelling kinetic studies were performed on printed disc specimens with a thickness of 2 mm and a diameter of 24 mm. For these studies, the change in sample thickness was monitored over time (5–300 min) to establish the point of swelling equilibrium. Dimensional measurements were taken immediately after sample removal from the annealing bath to avoid solvent evaporation. In addition, the mass and dimensions of all PP samples utilized in this study were monitored before immersion, immediately after removal from solvent, and after drying to ensure solvent removal and evaluate structural changes and recovery. Data were collected with a minimum of three separate measurements or replicates to calculate accurate average and standard deviation values.

Differential scanning calorimetry (DSC) experiments were performed utilizing a TA Instruments Discovery DSC 250 instrument with aluminum pans. Experiments were conducted under nitrogen using a heat-cool-heat cycle with a ramp rate of 5 °C/min from 40 to 160 °C, a cooling rate of 10 °C/min from 160 °C to −60 °C, and a final heating ramp of 5 °C/min from −60 to 160 °C. For annealed samples, the first heat thermogram was analyzed to determine the sample melting temperature (*T*_m_) and degree of crystallinity (*X*_c_). The first heat and cooling thermograms were then employed to calculate the lamellar thickness distribution of samples after immersion using the equation below

1 $$\frac{1}{M}\frac{dM}{dl}=\frac{dE}{dT}\frac{{\left(T_{m}^{0}-T_{m}\right)}^{2}\rho_{c}}{2\sigma_{e}T_{m}^{0}M}$$

where $\frac{1}{M}\frac{dM}{dl}$ represents the population of lamellae within a range of lamellar thicknesses that melt in a given temperature range, d*E*/d*T* represents the energy required to melt the lamellae, ρ_c_ is the density of the crystalline phase, and σ_*e*_ is the surface energy of the basal surface of a crystal.^50^

The cooling thermogram was also used to determine the crystallization temperature (*T*_c_). A cooling ramp from 40 °C to −60 °C was then employed to determine the glass transition temperature of PP printed samples before and after annealing. All data were analyzed using Trios software. DSC measurements were performed in triplicate to ensure accuracy, and average values and standard deviations were calculated. Thermogravimetric analysis (TGA) experiments were conducted using a TA Instruments Discovery TGA 550. Samples were heated with a ramp rate of 40 °C/min from room temperature to 600 °C under a nitrogen atomsphere.

Polarized optical microscopy (POM) images were collected with an Olympus BX53 M Upright LED Polarized Brightfield Darkfield microscope fitted with an Instec mK100 HCS302 temperature controller. Thin cross sections of unannealed and annealed tensile specimens were taken using a razor blade, placed on a microscope slide, and enveloped by a circular cover glass before being heated from ambient to 160 °C (above *T*_m_) at 10 °C/min. After holding samples at 160 °C for 10 min to ensure complete melting, pressure was applied to the cover glass until a thin film was formed to ensure image clarity, and an average sample thickness of 43 μm was achieved. Samples were then cooled to 90 °C at 10 °C/min and allowed to recrystallize under isothermal crystallization conditions for 5 min before being imaged. In situ sample imaging was performed by cooling the unannealed sample further to 50 °C at 10 °C/min before administering xylene. The sample was permitted to equilibrate for 5 min before micrographs were recorded. All micrographs were recorded using a Q-Imaging RoHS camera (2048 pixel × 1536 pixel) in the transmission mode at an angle configuration of 90° between the linear polarizer and the linear analyzer. Moreover, scanning electron microscopy (SEM) was performed utilizing a Zeiss Ultra 60 field-emission scanning electron microscope, employing an accelerating voltage of 21.0 kV.

Tensile properties of 3D-printed PP were determined following a modified ASTM D638 standard with a modified type I tensile bar.^51^ Generally, samples were printed with a nominal gauge length of 25 mm, a thickness of 4 mm, and a width of 12.5 mm and were examined using an MTS Insight mechanical testing frame equipped with a 2.5 kN load cell at a strain rate of 5 mm/min. Similarly, bulk PP type IV tensile bars were compression molded at 190 °C, with a nominal thickness of 0.3 mm and a width of 6 mm, and evaluated following ASTM D882 standards, with a strain rate of 5 mm/min, employing the same mechanical testing frame equipped with a 500 N load cell.^52^ Trouser tear specimens were printed in accordance with ASTM D624 standards to determine the fracture strength of specimens before and after expansion annealing.^53^ Samples were printed out-of-plane, with the initial tear being introduced by pausing midprint and taping a piece of paper, cut to the length dictated by the ASTM (40 mm), across the samples before resuming the print. An MTS Insight mechanical testing frame equipped with a 2.5 kN load cell and a strain rate of 50 mm/min was used for the tear test experiments. The fracture strength was calculated based on the load vs extension curves using the equation below

2 $$T=\frac{2F}{t}$$

where *T* represents the fracture strength, *F* represents normal force, and *t* represents the sample thickness. Data collection and analysis were performed using TestWorks and Igor Pro 9 software, respectively. All mechanical experiments were performed with a minimum of three samples to ensure an accurate calculation of the average properties as well as standard deviation values.

In situ small-angle X-ray scattering (SAXS) and wide-angle X-ray scattering (WAXS) measurements were performed using a Xeuss 3.0 system (Xenocs, Grenoble, France) equipped with a GeniX 3D beam delivery system, which provides monochromatic Cu Kα radiation. Scattering patterns were collected by using a Dectris Eiger 2R 1M-pixel 2D detector and reduced into an integrated 1D profile. The sample-to-detector distance was set to 0.045 m for WAXS and 0.9 m for SAXS measurements. For in situ annealing of SAXS experiments, small sections of unannealed tensile bar samples were soaked in *p*-xylene, sealed in 1.5 mm diameter capillaries, and loaded into a Linkam stage. The temperature was increased at a rate of 20 °C/min and held for 30 min or 1 h prior to recording measurements. The samples were exposed to the X-ray beam for 300 and 900 s for WAXS and SAXS measurements, respectively. Static SAXS measurements were performed at the 5-ID-D (DND-CAT) beamline of the Advanced Photon Source at the Argonne National Laboratory. A sample to detector distance of 8.5 m and an exposure time of 0.5 s were used for all measurements. Two-dimensional scattering patterns were collected with a MarMosaic 225 detector, azimuthally averaged, and reduced to one-dimensional data using software developed by beamline scientists at 5-ID-D.

## 3 Results and Discussion

PP was selected as the model system in this study since it is a common semicrystalline polymer used in MEX-AM, which exhibits a low glass transition temperature (*T*_g_) that permits sample deformability and elongation. Prior annealing approaches have largely focused on glassy, rigid polymers, whereas PP-based systems remain relatively underexplored. An overview of the expansion annealing mechanism is shown in Figure 1A. PP specimens were first printed by MEX in a continuous layer-by-layer process. Following printing, parts were immersed in xylene at controlled temperatures until they reached an equilibrium swelling state. The annealing temperature was varied to tune the degree of sample swelling and thus the extent of expansion of the amorphous domains, while the semicrystalline framework remained intact, thus preserving the structural integrity of samples throughout the annealing process. After removal from solvent, samples were dried and recovered to their original state; solvent evaporation and cooling triggered chain recrystallization and intermolecular entanglements across adjacent layers (such as tie chain formation), leading to enhanced interlayer adhesion in MEX-printed PP parts. We note that there are potential environmental and safety concerns regarding the use of xylene as a solvent system for expansion annealing of PP; therefore, future studies exploring alternative, less hazardous solvent systems will be needed. Importantly, this study serves as a fundamental experimental platform that can be adapted to other MEX-AM semicrystalline polymer materials or solvent systems.

**Figure 1:**
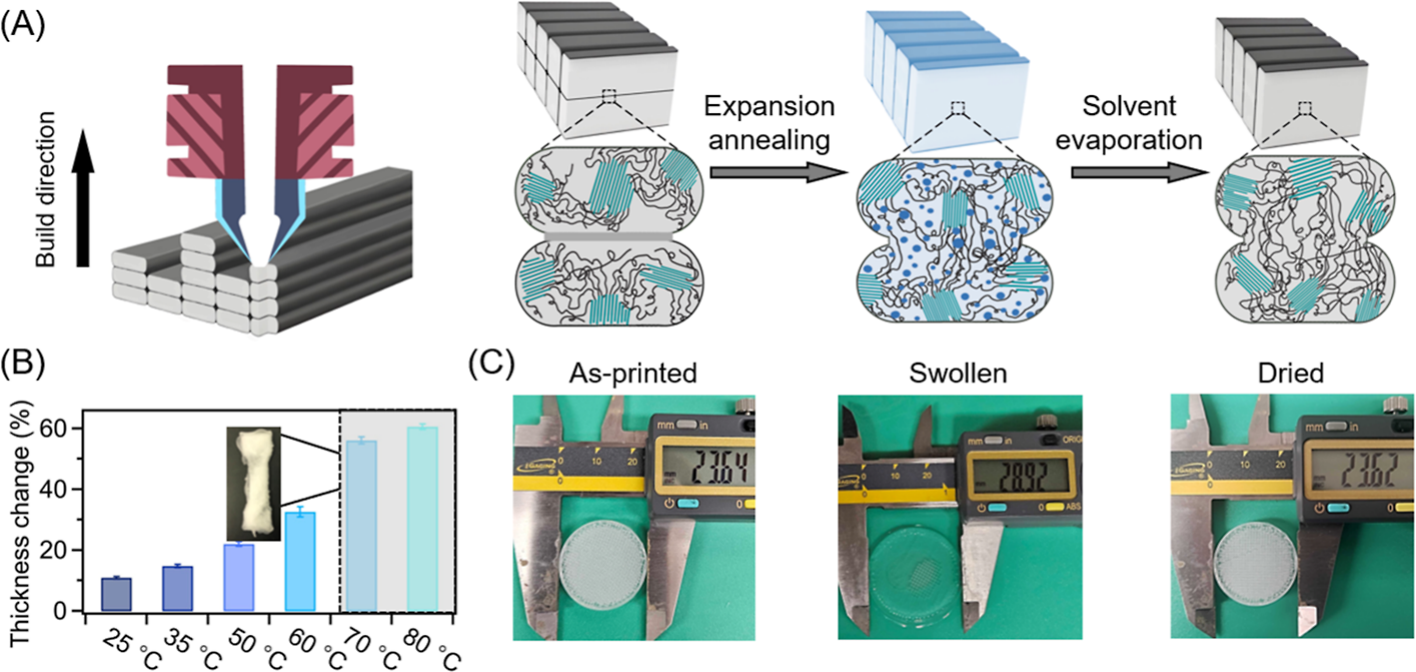
(A) Illustration of expansion annealing process. (B) Average swelling equilibrium thickness change for temperatures ranging from 25 to 80 °C. (C) Images displaying PP-printed disc samples before expansion annealing, immediately after immersion, and after complete drying.

We first investigated the macroscopic swelling behavior of the printed PP parts under different annealing temperatures in xylene by monitoring the dimensional changes of printed disc-shaped specimens (a model sample geometry). When annealed at temperatures ranging from 25 to 60 °C, samples achieved an equilibrium swelling state within ∼100 min (Figure S1). As shown in Figure 1B, the extent of sample expansion at equilibrium increased with temperature, reaching 10.5% at 25 °C and 31% at 60 °C. However, at annealing temperatures above 60 °C, significant sample distortion was observed due to swelling-induced stress, which compromised structural integrity. As a result, dimensional measurements became unreliable beyond approximately 70 min for samples annealed at 70 and 80 °C. In our process, the retained crystallites during annealing act as physical cross-links for preserving part integrity. If the crystallites are too small, or if the stress generated by amorphous phase expansion becomes excessive, these physical cross-links are unable to maintain the structure, thereby preventing recovery to the original shape. Based on these observations, subsequent experiments were limited to expansion annealing temperatures within the range of 25 to 60 °C to ensure controlled swelling and retained structural integrity of the PP specimens. We note that dimensional measurements of the disc specimens at each temperature confirmed near-isotropic swelling, with less than 2% variation across both sample dimensions. After complete drying, the specimens recovered to their original dimensions and mass, being nearly identical to the neat state (Figure 1C). This result confirms that expansion annealing can be used to engineer the properties of printed parts (post-AM) while fully retaining their macroscopic structure.

The expansion annealing process was then applied to samples of varying thicknesses, as well as bulk samples at 50 °C to evaluate the influence of the critical sample dimension and structure on the swelling behavior of printed specimens. Geometries examined included step-like printed samples with thicknesses ranging from 2 to 15 mm, as well as compression-molded and 3D-printed rectangular prisms (with average thicknesses of ∼0.6 mm) (Figure S2A,B). For the step-like samples, all regions swelled uniformly and reached equilibrium after ∼100 min regardless of thickness, with less than 3% variation in thickness change. Similarly, both compression-molded and printed prisms reach equilibrium after ∼50 min, with a dimensional variation of only ∼3%. These results indicate that the swelling behavior of PP is governed by both the diffusion of xylene through the amorphous phase and the inherent porosity generated within MEX-AM parts. As shown in Figure S3, these samples inherently contain porosity between printed filament layers, which facilitates solvent penetration and contributes to consistent swelling behavior across different thicknesses. This characteristic feature of expansion annealing is critical in demonstrating the scalability of our process to samples with larger feature sizes.

In situ small-angle X-ray scattering (SAXS) and wide-angle X-ray scattering (WAXS) were performed on model samples immersed in xylene at temperatures ranging from 25 to 70 °C to understand how the annealing process affected the mesoscale morphology of our samples. For all samples, regardless of annealing temperature, Lorentz-corrected SAXS profiles show a primary scattering peak, corresponding to the long period (*L*), indicating that the PP material remained in its semicrystalline state during the annealing process. The position of the primary scattering peak (q*) shifted to a lower region as annealing temperatures increased, indicating a significant expansion of the amorphous phase (Figure 2A). Specifically, the neat sample displayed an *L* value of 11.3 nm in the dry state. After immersion in xylene at 35, 50, and 60 °C, *L* increased to 16.0, 18.1, and 19.6 nm, corresponding to a 42, 60, and 73% increase, respectively, compared to the neat state (Figure 2B). The increase in *L* at higher annealing temperatures is attributed to partial melting of crystalline lamellae, which reduces the sample crystallinity, enabling the larger amorphous fraction to permit a greater swelling degree. WAXS results displayed characteristic diffraction peaks indicating that the PP material existed in its monoclinic form, as shown in Figure 2C.^54,55^ These peaks persist across all annealing temperatures examined, confirming that the expansion annealing process does not disrupt the unit-cell structure of PP crystalline domains. Moreover, polarized optical microscopy (POM) was used to characterize PP crystalline spherulites before, during, and after expansion annealing (Figure S4). Upon exposure to xylene at 50 °C, birefringence intensity decreased, indicating a reduced crystalline fraction consistent with partial lamellar melting (Figure S4A,B). After drying, birefringence recovered as a result of PP chain recrystallization (Figure S4C). These observations align with scattering results showing a preserved semicrystalline morphology during the annealing process and temperature-dependent expansion of amorphous regions in PP samples.

**Figure 2:**
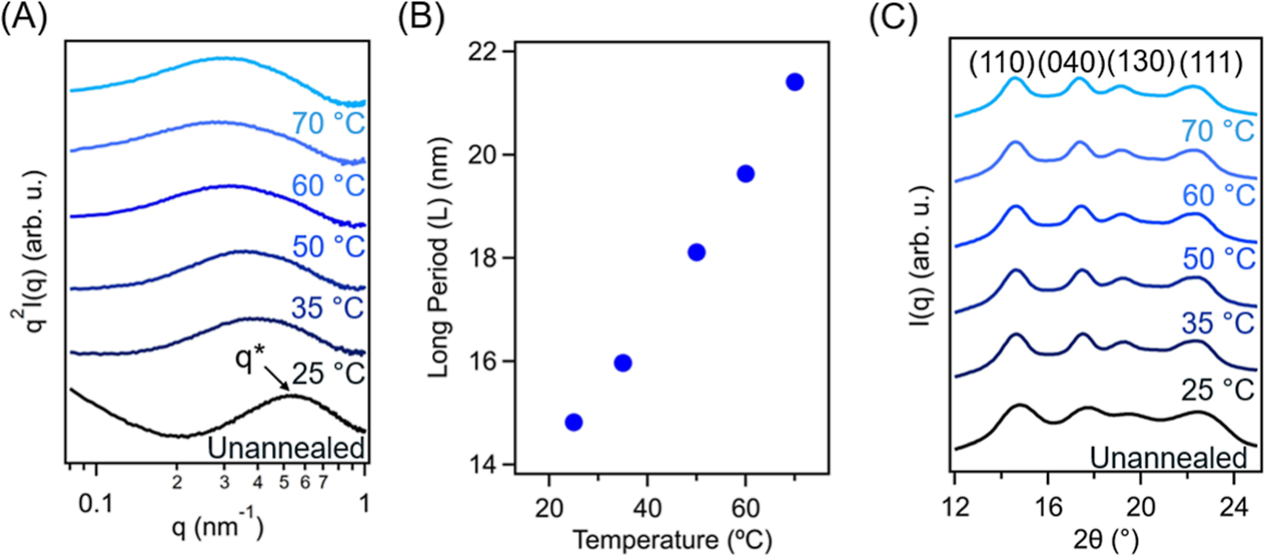
(A) In situ, Lorentz-corrected small-angle X-ray scattering (SAXS) profiles of PP immersed in xylene at temperatures ranging from 25 to 70 °C. (B) Long period (*L*) as a function of immersion temperature. (C) In situ wide-angle X-ray scattering (WAXS) of PP annealed at temperatures ranging from 25 to 70 °C.

To understand the impact of expansion annealing on sample mechanical properties, dog-bone specimens were printed (following a modified ASTM D638 standard) with a build direction along the out-of-plane orientation (*z*-axis; Figure 3A). All samples underwent a standardized annealing process: immersion in xylene for 150 min at 25, 35, 50, or 60 °C, followed by cooling to room temperature prior to removal and vacuum drying at 40 °C for 48 h. Additionally, solvent removal was confirmed by thermogravimetric analysis (TGA), in which the annealed tensile specimen showed essentially identical mass change behavior to that of the unannealed specimen (Figure S5). Tensile tests were then performed along the *z*-direction, applying a load normal to the layer deposition direction. As shown in Figure 3B, the unannealed specimen is relatively brittle, exhibits minimal yielding, and fails at <50% elongation. After immersion in xylene at 50 and 60 °C, the sample mechanical response changes substantially (Figure 3A). These annealed specimens show a clear yield point, modest strain hardening behaviors, and ductile failure; we note that expansion annealing at 25–35 °C produced limited improvements, which suggests a critical swelling condition is required to promote interlayer adhesion in these PP parts. Figure 3C quantifies mechanical property improvements as a function of the expansion annealing temperature. Annealing at elevated temperatures dramatically enhanced material toughness, increasing from 3.57 MJ/m^3^ for unannealed specimens to 6.2, 6.7, 25, and 22 MJ/m^3^ after annealing at 25, 35, 50, and 60 °C, respectively. Elongation at break values also greatly improved, rising from 46% to 82, 93, 330, and 290% when immersed at 25, 35, 50, and 60 °C, respectively. The considerable improvements observed in sample ductility after annealing at 50 and 60 °C are attributed to amorphous phase expansion in conjunction with partial lamellar melting during annealing, which allows for the formation of interlayer entanglements and an increased number of tie chain molecules upon recrystallization, resulting from solvent removal and sample cooling. It should be noted that the mechanical properties of these annealed specimens remain lower than those of the bulk material. Furthermore, the limited ductility improvements observed after annealing at 25 and 35 °C suggest that the temperature is a key parameter governing the extent of sample expansion and crystalline domain melting, which in turn dictates the properties of the annealed samples. Moreover, for MEX-AM printed PP specimens, plasticization effects resulting from xylene exposure alone would not be expected to improve the mechanical performance. As PP is a low *T*_g_ material, applied stress during tensile testing predominantly leads to chain mobility and slippage, and thus plasticization alone cannot account for the observed mechanical enhancement. Instead, the improvement in mechanical properties is attributed to the formation of tie chains and enhanced crystallization across interlayer regions, and these effects will be discussed in detail in the following section. Reductions in both tensile strength and modulus were observed for annealed specimens compared to the as-printed state. The tensile strength of samples annealed at 25, 35, 50, and 60 °C decreased from 9.0 MPa to 8.7, 8.1, 8.2, and 8.1 MPa (a decrease of 3.3, 9.7, 8.8, and 9.7%, respectively). Modulus values also decreased with increasing annealing temperature, diminishing from 220 MPa to 194, 166, 133, and 136 MPa after annealing at 25, 35, 50, and 60 °C, respectively (Figure S6). We believe the decrement in tensile strength and modulus values results from crystal restructuring upon our annealing treatment.^56,57^ Overall, the significant improvement in elongation at break and toughness of printed PP parts confirms the enhanced interlayer adhesion after annealing at elevated temperatures. To corroborate this, we performed trouser tear tests (ASTM D624) on out-of-plane printed specimens. Average fracture strength values of samples annealed at 25 °C were comparable to the neat sample, but they increased substantially with higher annealing temperatures, showing an improvement of 24% at 35 °C, 47% at 50 °C, and 75% at 60 °C (Figure 3D), further demonstrating enhanced interlaminar bonding via expansion annealing. These results, together with the tensile tests, confirm that our method strengthens the interlayer adhesion via sample swelling and deswelling. Specifically, the improvement stems from amorphous domain expansion during annealing, which can promote interlayer chain interactions. Additionally, it is found that mechanical performance enhancements increase with the degree of sample swelling, and thus, a minimum dimensional expansion threshold is required to achieve meaningful improvements.

**Figure 3:**
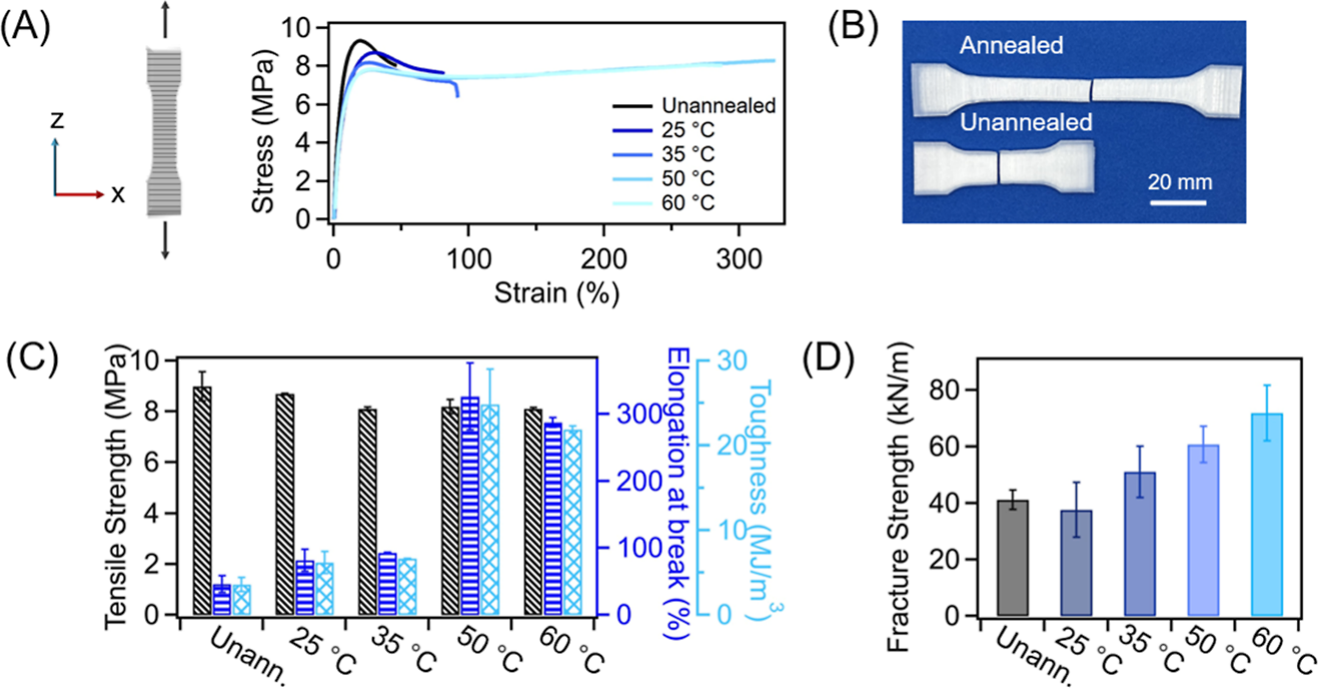
(A) Illustration demonstrating printing orientation of out-of-plane printed tensile bars and representative stress vs strain curves for unannealed and annealed samples, immersed for 150 min at temperatures ranging from 25 to 60 °C. (B) Image displaying unannealed and annealed tensile bars post-fracture. (C) Tensile properties of unannealed and annealed PP, including tensile strength, elongation at break, and toughness. (D) Fracture strength of unannealed and annealed specimens.

While prior studies have shown that increased chain entanglement can improve the interlayer strength and mechanical response of MEX-printed parts, most focused on glassy, rigid polymers, where chain entanglement dominates the sample mechanical performance.^58^ In our system of PP, which exhibits low *T*_g_, entanglements alone are insufficient, as polymer chains could be pulled and disentangled easily under a tensile load. In commodity polyolefins, mechanical response is governed by crystalline morphology and tie chain formation.^59,60^ Accordingly, the ability of our annealed samples to sustain load transfer across distinct layers is attributed to their semicrystalline microstructure. To further elucidate the mechanistic origin of the mechanical property improvements from expansion annealing, differential scanning calorimetry (DSC) analysis (Figure S7) of neat and annealed PP specimens was performed to understand their thermal properties, such as the degree of crystallinity (*X*_c_), crystallization temperature (*T*_c_), and glass transition temperature (*T*_g_). Neat PP exhibits an *X*_c_ of approximately 19.4%, which remains essentially unchanged after annealing at 25 °C. Annealing at higher temperatures increases sample crystallinity, reaching 20.1%, 20.5%, and 22.1% after immersion at 35, 50, and 60 °C, respectively (Figure 4A). Moreover, the *T*_c_ (Figure 4A) of these PP specimens also increases with expansion annealing temperature, from 94.9 °C (neat) to 95.2 and 95.8 °C after immersion at 50 and 60 °C, respectively. These trends indicate that upon xylene removal, polymer chains can recrystallize, and enhanced interlayer interactions/entanglement formation during the solvent-induced expansion state facilitates additional crystal formation across adjacent layers within the printed parts. The higher *T*_c_ in annealed samples suggests the samples crystallize more readily, likely due to improved chain organization and interlayer entanglements that facilitate nucleation and lamellar growth. It should also be mentioned that the *T*_g_ of the printed PP slightly changed from −27.3 °C for the unannealed sample to −31.0, −30.9, −27.3, and −27.8 °C after annealing at 25, 35, 50, and 60 °C, respectively, indicating the absence of plasticization of the amorphous domain at higher annealing temperatures (Figure S7).

**Figure 4:**
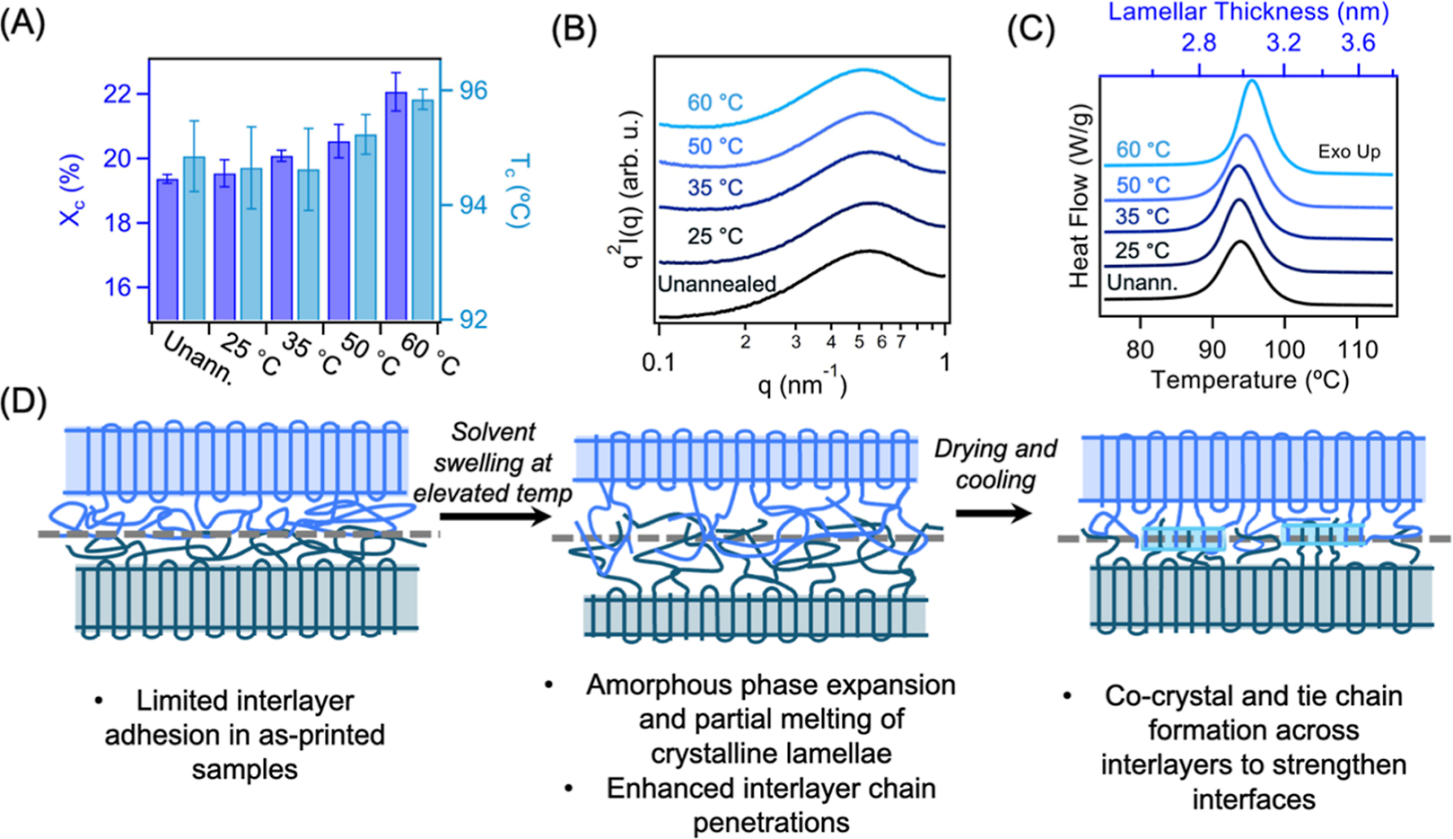
(A) Degree of crystallinity and crystallization temperature of PP samples before and after annealing at various temperatures. (B) Static Lorentz corrected SAXS profiles of unannealed and completely dried, annealed PP samples. (C) Lamellar thickness distributions of unannealed and annealed single filament traces, based on cooling thermograms. (D) Illustration highlighting the proposed mechanism for interlayer chain interpenetration during immersion and cocrystallite and tie chain formation upon solvent removal.

The crystallization behavior of dual- and single-layered specimens was then investigated to distinguish the effect of expansion annealing on 3D printed as well as bulk PP specimens, respectively. As shown in Figure 4B, SAXS results of dried PP samples annealed between 25 and 60 °C show a small variation of *L* less than 0.6 nm relative to the unannealed neat sample, indicating near complete recovery of the amorphous domain after xylene removal, consistent with the observed macroscopic return to original dimensions. Lamellar thickness distributions of the single-layered specimens, created by utilizing the cooling thermogram, display a shift toward larger crystallites with an increasing immersion temperature (Figure 4C). Unannealed samples exhibited a peak position at 2.97 nm. After annealing at 25 °C, this peak position shifts slightly to lower thickness values, with a peak position located at 2.96 nm (we note the lamellar thickness distribution of samples annealed at 35 °C possessed a similar peak position to the neat sample ∼2.97 nm). Upon immersion at 50 and 60 °C, however, the peak positions shift toward slightly larger thicknesses, with values of approximately 3.00 and 3.03 nm, respectively. Lamellar thickness distributions of the dual-layered specimens, based on first-heat melting thermograms, display a similar trend with small shoulders appearing after annealing at elevated temperatures (Figure S8). The appearance of these shoulders indicates a new crystalline melting event that shifts to higher temperatures with increasing annealing temperature. Specifically, the shoulders display peak positions of 3.24 and 3.36 nm after annealing at 50 and 60 °C, respectively. This crystallization behavior further suggests that expansion annealing promotes the formation and growth of interlayer crystallites across distinct printing layers, thereby enhancing mechanical properties along the out-of-plane direction (Figure 4D). Specifically, during annealing, solvent-induced expansion at an elevated temperature allows polymer chains from adjacent layers to interpenetrate across interfacial boundaries. As the solvent is removed and the system recrystallizes, these interpenetrated chains can form cocrystals or tie chains that bridge previously discrete layers, which effectively reduce interfacial defects and improve load transfer between layers. As a result, the typically weak interlayer adhesion inherent in MEX-AM structures is significantly strengthened. The formation of these interlayer crystallite structures and tie-chains is responsible for the improved structural integrity and higher extensibility of printed samples.

To study how print orientation affects the efficacy of expansion annealing for improving the mechanical properties of PP, tensile specimens were prepared with orientations ranging from in-plane (0°), 30°, 45°, and 60° (with respect to the *x*-axis) and compared to the results out-of-plane (90°) annealed and unannealed samples (as well as bulk, compression molded PP type IV ATSM D638 tensile bars, with properties indicated by the red dashed line in Figure 5A–C). In these experiments, all samples were annealed in xylene at 50 °C for 150 min and then dried under vacuum to remove residual solvent. An illustration of the tensile specimen MEX-AM print orientation is shown in Figure 5D. For 0° printed specimens, expansion annealing caused slight reductions in tensile strength (−7.2%), toughness (−20%), and elongation at break (−19%), compared to the neat sample (Figure 5A–C). The decrement in properties observed for these specimens arises because they were evaluated with a load parallel to the filament deposition orientation; thus, improved interlayer adhesion has little impact on their tensile response. Instead, another factor likely dominates: disruption of printing-induced chain alignment. Expansion annealing promotes chain expansion, relaxing the amorphous network from a mildly aligned state toward a more isotropic configuration. This can reduce load transfer along the filament axis, even as crystallinity increases. However, as the tensile orientation shifted toward a 90° orientation (30, 45, and 60°), the properties of the annealed specimens gradually improved, eventually approaching or surpassing bulk properties (Figure 5A–C). Specifically, specimens with a 30° orientation experienced a slight increase in tensile strength, toughness, and elongation at break from 11.9 MPa, 76.6 MJ/m^3^, and 781% (for unannealed, 3D printed specimens) to 12.1 MPa, 83.1 MJ/m^3^, and 865% upon annealing (increasing by 0.7%, 7.6%, and 10%), respectively. We note that samples with a 45° and 60° printing orientation exhibited significant improvements, with tensile strength improving from 13.2 and 11.4 MPa, for unannealed, MEX-printed samples, to 14.5 and 13.4 MPa, respectively; toughness improving from 87.7 and 64.2 MJ/m^3^ to 137 and 91.7 MJ/m^3^, respectively; and elongation at break improving from 839 and 671% to 1230 and 864%, respectively. Notably, tensile properties of annealed samples printed with a 45° orientation exceeded bulk tensile properties, with toughness increasing from 98.6 MJ/m^3^ to 137 MJ/m^3^ and elongation at break improving from 658% to 1230%. Moreover, printed samples with this orientation possessed greater tensile properties relative to other printing orientations, as the load applied during tensile experiments was shared and effectively dissipated by both interlaminar welds and individual filament traces.^61^ Furthermore, unlike bulk PP, which exhibits a tendency for localized deformation, the 45° raster configuration could promote printed filament rotation toward the loading direction under tensile stress. This reorientation delays strain localization and facilitates a more uniform stress redistribution. As a result, improved interlayer adhesion enables these structures to achieve, and in some cases surpass, bulk PP performance, particularly in terms of toughness and elongation at break. To evaluate whether the bulk samples exhibited a crystalline structure comparable to that of the MEX-AM-printed specimens, both compression-molded and printed PP materials were analyzed and compared through DSC experiments (Figure S9). Compression molded samples experienced a slight decrease in *X*_c_ and *T*_c_ values relative to the MEX-AM PP, with *X*_c_ decreasing from 19% to 17% and *T*_c_ decreasing from 94 to 93 °C. This change is likely attributed to different sample cooling histories and the alignment of PP chains during the MEX-AM process, which promotes improved polymer crystallization behavior.^62^ Furthermore, the improvements shown by samples oriented closer to the out-of-plane (*z*) direction represent the advantages gained by employing expansion annealing, as the improved interlayer adhesion further enhances load transfer across interlaminar interfaces and yields superior tensile properties.

**Figure 5:**
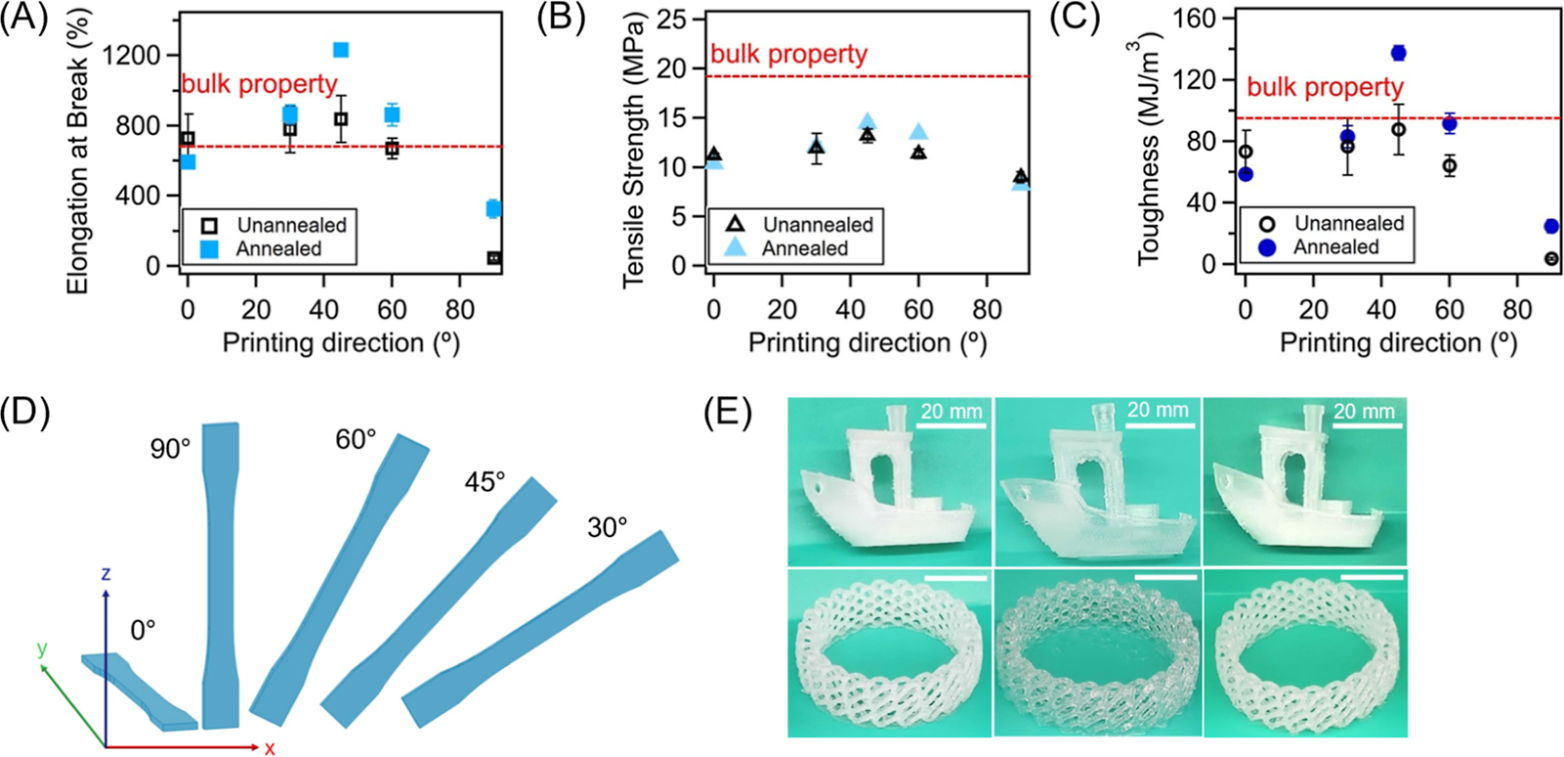
Tensile properties as a function of printing orientation compared to bulk, compression-molded samples; properties include (A) elongation at break, (B) tensile strength, and (C) toughness. (D) Image exhibiting the different printing orientations utilized for this study. (E) Complex structures before expansion annealing, immediately after immersion, and after complete drying.

The applicability and versatility of the expansion annealing method can be confirmed by demonstrating geometrically complex prints, including a standard Benchy and an open-celled disc. Both parts were subjected to annealing in xylene at 50 °C for 150 min, followed by a complete drying process. Figure 5E displays recorded images of printed parts pre-immersion, immediately post-immersion, and post-drying using identical camera settings. Upon immersion, the disc and boat structures exhibited swelling of ∼20% and ∼15% in characteristic lengths, respectively (outer diameter and height for the disc; overall length for Benchy). Critically, after solvent removal and drying, each structure returned to its original dimensions within <1% deviation, with no noticeable warpage or feature collapse. These results indicate that expansion annealing can be implemented on complex, nonprismatic architectures without sacrificing dimensional fidelity, attributed to the retention of the semicrystalline framework during expansion annealing.

## 4 Conclusions

In conclusion, this work addresses a central bottleneck in MEX-AM, associated with poor interlayer adhesion within printed parts, by developing expansion annealing as a post-manufacturing annealing method. Upon immersing printed PP parts within xylene at elevated temperatures, the solvent penetrates and selectively swells the amorphous phase to promote interlaminar chain interaction and entanglement formation, while the retention of crystalline lamellae aids in maintaining part geometry. After drying, chains recrystallize across interfaces, forming interfacial crystallites (i.e., cocrystals) and tie chains that markedly strengthen interlayer adhesion. Our method requires no special equipment or custom filaments, preserves the semicrystalline microstructure, and can be adapted to complex geometries—offering a simple, scalable, and drop-in annealing process that strengthens interlayer adhesion post-AM.

## Supplementary Material



## Notes

Graph of thickness change versus time for expansion-annealed PP discs; graph displaying thickness change as a function of time for compression molded and 3D printed rectangular prisms; graph of thickness change over time for step-like specimens with varied thicknesses; SEM micrograph of the cross-section of an annealed tensile bar; polarized optical micrographs of PP before, during, and after annealing; TGA curves of annealed and unannealed tensile bar specimens; bar graph displaying change in modulus with annealing temperature; DSC first heat of dual-layered specimens; plot showing change in *T*_g_ with annealing temperature; and bar graph comparing the differences in the crystalline structure of MEX-AM printed and compression molded PP (PDF)
