# Supplementary material for: Welding Adjacent Layers in Additively Manufactured Polypropylene via Expansion Annealing
Source: Macromolecules. 2026 Apr 27;59(9):5461–70. doi: 10.1021/acs.macromol.6c00263 (PMC13173652; doi:10.1021/acs.macromol.6c00263)
Supplement: Supplementary file 1 [file ma6c00263_si_001.pdf]

Supporting Information For:

Welding adjacent layers in additively manufactured polypropylene via expansion annealing

Zoe Gunter[1], Anthony Griffin[1], Niyati Tamang[1], and Zhe Qiang[1,*]

[1] School of Polymer Science and Engineering, University of Southern Mississippi, *118 College Drive, Hattiesburg, Mississippi 39406, United States*

[*]Corresponding Author: Z. Q. (Email: zhe.qiang@usm.edu)

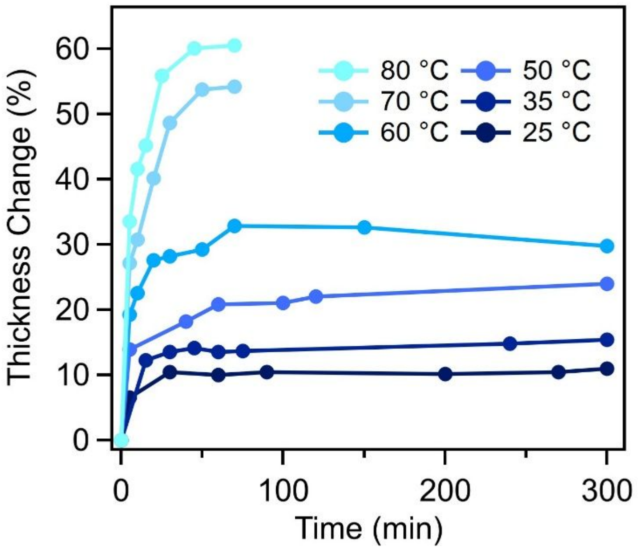

Figure S1. Thickness change as a function of time for polypropylene disks annealed in xylene at temperatures ranging from 25 to 80 °C.

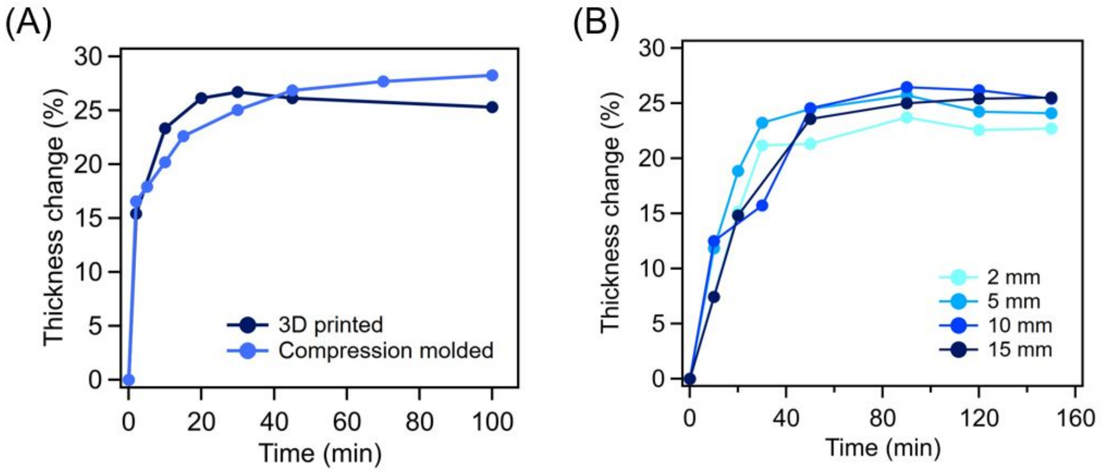

Figure S2. Thickness changes as a function of time for A) compression molded and 3D printed rectangular prisms with a thickness of approximately 0.6 mm and B) step-like specimens with thicknesses ranging from 2 to 15 mm; samples were immersed in xylene at 50 °C for 150 min.

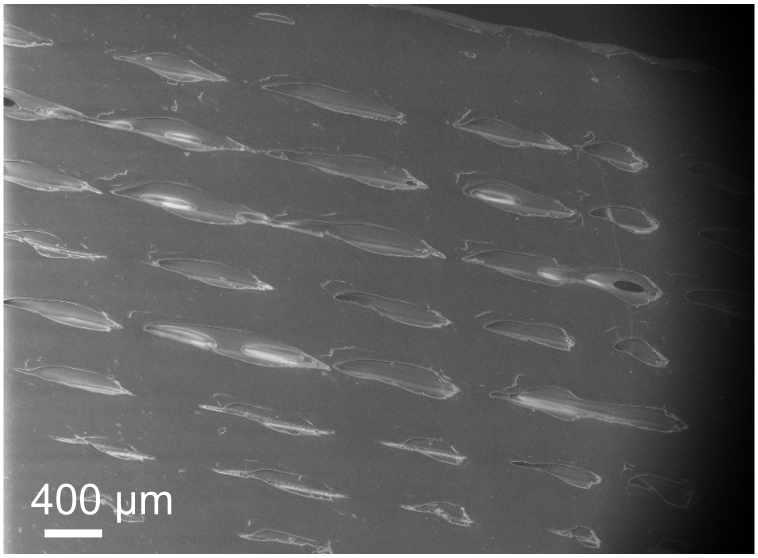

Figure S3. SEM micrograph of the cross-section of a MEX-AM printed tensile bar (after annealing at 50 °C for 150 min and vacuum drying for 48 h at 40 °C).

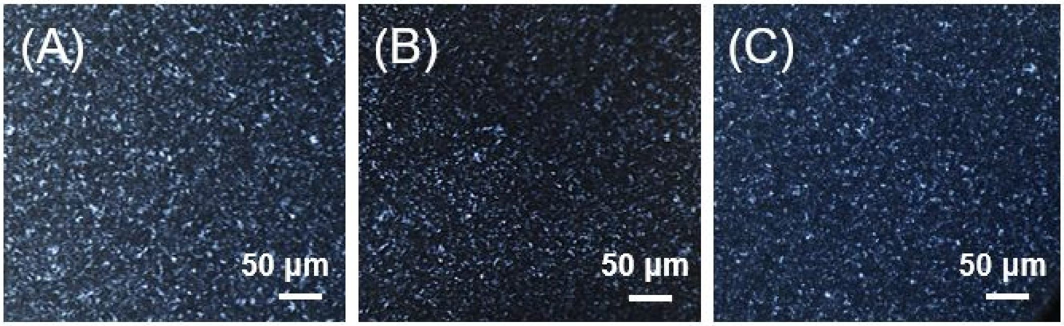

Figure S4. POM micrographs of (A) unannealed PP, (B) in situ annealed PP (annealed at 50 °C in xylene), and C) annealed PP after drying (micrographs recorded with an angle configuration of 90° between the linear polarizer and the linear analyzer).

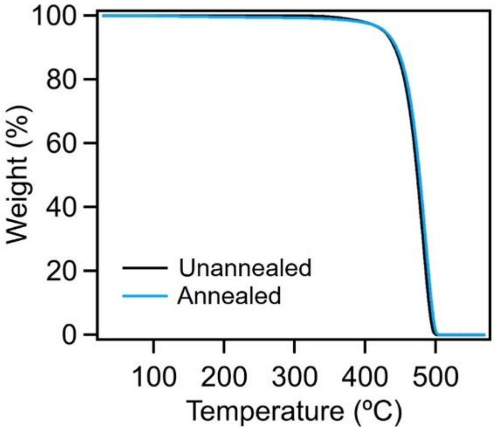

Figure S5. TGA curves of an unannealed (black) and annealed (blue) tensile bar, which was annealed at 50 °C for 150 min and vacuum dried for 48 h at 40 °C.

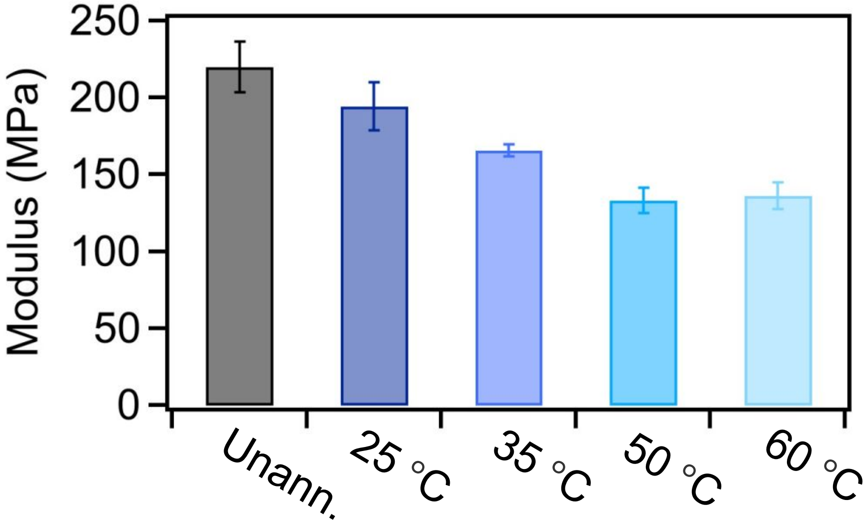

Figure S6. Tensile modulus values for unannealed and annealed samples; all samples were immersed for 150 min in xylene and dried for 48h at 40 °C.

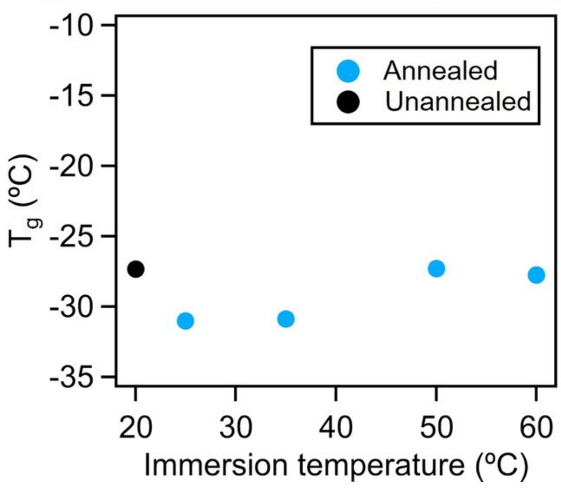

Figure S7. Glass transition temperatures for unannealed and annealed PP (annealed for 150 min and dried prior to measurement) based on a DSC cooling ramp.

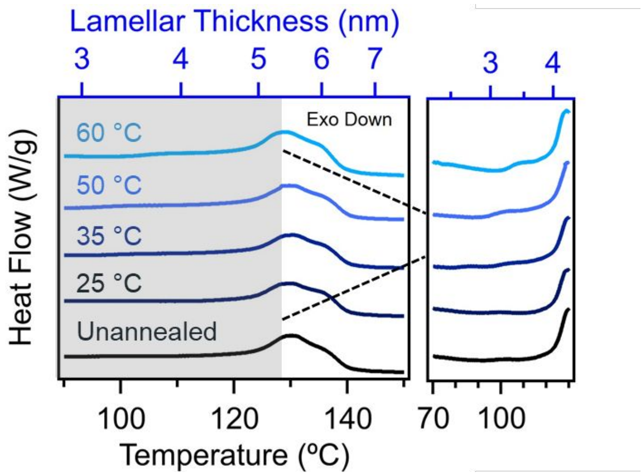

Figure S8. Lamellar thickness distributions based on first-heat DSC thermograms of dual-layered filament traces annealed for 150 min at temperatures ranging from 25 to 60 °C, with a shoulder region highlighting the lamellar thickness distributions in the temperature range of 70 to 130 °C.

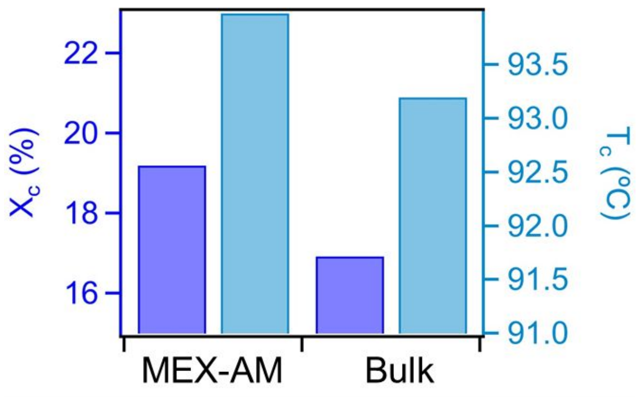

Figure S9. Degree of crystallinity and crystallization temperature of MEX-AM and compression molded PP specimens.
